# Charge-Mediated Co-assembly of Amphiphilic Peptide and Antibiotics Into Supramolecular Hydrogel With Antibacterial Activity

**DOI:** 10.3389/fbioe.2020.629452

**Published:** 2020-12-23

**Authors:** Lei Xu, Qian Shen, Linzhuo Huang, Xiaoding Xu, Huiyan He

**Affiliations:** ^1^Guangdong Provincial Key Laboratory of Malignant Tumor Epigenetics and Gene Regulation, Medical Research Center, Sun Yat-sen Memorial Hospital, Sun Yat-sen University, Guangzhou, China; ^2^Department of Clinical Pharmacy, The Second Affiliated Hospital, University of South China, Hengyang, China; ^3^Central Sterile Supply Department (CSSD), Sun Yat-sen Memorial Hospital, Sun Yat-sen University, Guangzhou, China

**Keywords:** amphiphilic peptide, self-assembly, supramolecular hydrogel, sustained release, antibacterial activity

## Abstract

Bacteria are the most common pathogens to cause infection of surgical sites, which usually induce severe postoperative morbidity and more healthcare costs. Inhibition of bacteria adhesion and colonization is an effective strategy to prevent the spread of infection at the surgical sites. Hydrogels have been widely used as promising antibacterial materials, due to their unique porous structure that could accommodate various antibacterial agents (e.g., antibiotics and cationic polymers with inherent antibacterial activity). Herein, inspired by the natural protein self-assembly, an amphiphilic peptide comprised of a hydrophobic naphthyl (Nap) acetyl tail and a hydrophilic peptide backbone was employed to construct supramolecular hydrogel for sustained release of the antibiotic polymyxin B. At neutral pH, the negatively charged amphiphilic peptide could form electrostatic attraction interaction with the positively charged polymyxin B, which could thus drive the ionized peptide molecules to get close to each other and subsequently trigger the self-assembly of the amphiphilic peptide into supramolecular hydrogel *via* intermolecular hydrogen bonding interaction among the peptide backbones and π-stacking of the hydrophobic Nap tails. More importantly, the electrostatic attraction interaction between polymyxin B and the amphiphilic peptide could ensure the sustained release of polymyxin B from the supramolecular hydrogel, leading to an effective inhibition of Gram-negative bacteria *Escherichia coli* growth. Combining the good biocompatibility of the amphiphilic peptide, the supramolecular hydrogel developed in this work shows a great potential for the surgical site infection application.

## Introduction

Surgical site infections are persistent and severe issues that prevent wound healing and induce wound dehiscence, abscess formation, and sepsis (Schierholz and Beuth, 2001; Edwards and Harding, 2004; Owens and Stoessel, 2008). According to the Center for Disease Control and Prevention (CDC), the ratio of surgical site infections reaches ∼22% of total hospital-related infections (Magill et al., 2014). Bacteria are the most common pathogens that induce surgical site infections. Once the bacteria adhere to the surgical sites and proliferate to form biofilm, bacteria could effectively escape from killing by antibiotics or eliminating by body immune system (Costerton et al., 2005; Wagner and Hänsch, 2017; Arciola et al., 2018). Therefore, the key point to prevent the spread of infection at the surgical sites is to inhibit bacteria adhesion and colonization (Mah and O’toole, 2001). In the past decade, intensive efforts have been focused on the design and development of antibacterial materials, including antibiotics, quaternary ammonium compounds, metal ions or particles, cationic polymers, and antibacterial peptides (Arciola et al., 2012; Campoccia et al., 2013; Modaresifar et al., 2019). Although these developed materials show good antibacterial characteristic *via* destroying bacterial membrane or disrupting bacterial functions, short drug effect duration and potential hemolytic activity may limit their wide application (Tian et al., 2020).

In recent years, hydrogels have emerged as a promising platform to construct antibacterial materials, due to their porous structure and easily tailored functionality (e.g., pore size and mechanical strength) (Lee et al., 2013; Li et al., 2018; Zhao et al., 2020). It is known that hydrogels have a three-dimensional network at hydrated environment, which is extremely similar to the natural extracellular matrix (ECM) and could thus maintain the host cell function to promote wound healing. Unfortunately, the hydrated environment of hydrogels could also improve the proliferation of bacteria adhered to the surgical sites (Schierholz and Beuth, 2001). Therefore, antibacterial agents such as antibiotics, metal particles, and cationic polymers with inherent antibacterial capability are usually incorporated into hydrogel networks to endow them with antibacterial activity (Li et al., 2018; Fan et al., 2020; Zhao et al., 2020). In the past few years, several polymers with bacteria membrane-destroying characteristic have been also developed to construct antibacterial hydrogels to inhibit surgical site infections (Salick et al., 2009; Liu et al., 2012; Giano et al., 2014; Wang et al., 2019). From the standpoint of clinical translation, because the complicated synthesis strategy may introduce difficulty in scale-up of the hydrogels made with antibacterial polymers, direct incorporation of antibacterial agents into hydrogels may be more advantageous. In addition, it is more convenient to adjust biocompatibility and antibacterial activity of the hydrogels loading antibacterial agents *via* simply changing their compositions (Lee et al., 2013). Especially for the supramolecular hydrogels self-assembled from peptides, due to their unique advantages such as wide availability of sources and inherent biodegradability, peptide hydrogels have been widely used for drug delivery, tissue engineering, and would healing (Ulijn and Smith, 2008; Koutsopoulos, 2016; Lu and Wang, 2018). Moreover, compared to many polymeric hydrogels with complicated synthesis strategy, the facile peptide synthesis method (e.g., solid phase synthesis technique) enables the scale-up of peptide hydrogels for various applications.

**FIGURE 1:**
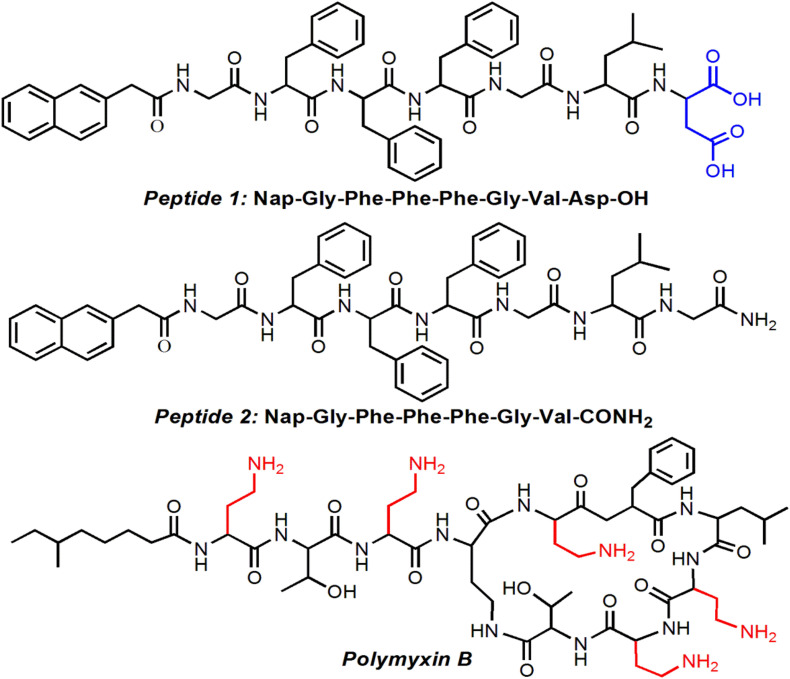
Chemical structures of the amphiphilic peptides and polymyxin B.

Along the principles described above, we herein designed and developed a new amphiphilic peptide that could co-assemble with polymyxin B, a widely used antibacterial agent for inhibition of Gram-negative bacterial infections (Tsubery et al., 2000; Bhor et al., 2005), to form well-defined supramolecular hydrogel. This amphiphilic peptide is comprised of a hydrophobic Nap tail and a hydrophilic peptide backbone (Figure 1). In aqueous solution at neutral pH, the negatively charged amphiphilic peptide could form electrostatic attraction interaction with the positively charged polymyxin B, which could thus drive the ionized peptide molecules to get close to each other and subsequently trigger the self-assembly of the amphiphilic peptide into stable hydrogel. More importantly, this electrostatic attraction interaction could ensure the sustained release of polymyxin B from the supramolecular hydrogel, thereby achieving an effective inhibition of Gram-negative bacteria *E. coli* growth.

## Materials and Methods

### Materials

Polymyxin B sulfate salt, calcein AM, ethidium homodimer-1 (EthD-1), dimethyl sulfoxide (DMSO), sodium hydroxide (NaOH), and hydrochloric acid (HCl, 37 wt%) were acquired from Sigma-Aldrich and used directly. Fluorescein isothiocyanate was provided by Thermo Fisher and used as received. Two amphiphilic peptides (Nap-Gly-Phe-Phe-Phe-Gly-Val-Asp-OH and Nap-Gly-Phe-Phe-Phe-Gly-Val-CONH_2_) were designed according to our previously studies (Xu et al., 2011, 2012) and synthesized by GL Biochem Ltd. (Shanghai, China). Phosphate buffered saline (PBS) solution, Dulbecco’s Modified Eagle’s Medium (DMEM), luria broth (LB) medium, fetal bovine serum (FBS), penicillin-streptomycin, trypsin, and were provided by GIBCO Invitrogen Co. All other reagents and solvents were of analytical grade and used directly.

### Preparation of Peptide Hydrogel

The amphiphilic peptide (5 mg) was suspended in 1 mL of deionized (DI) water and a small volume of NaOH aqueous solution (1 M) was added to adjust the solution pH to a basic pH (∼10) to ensure the formation of homogeneous peptide solution. Subsequently, concentrated HCl aqueous solution (6 M) was added in 1–3 μL increments to adjust the solution pH and a stable hydrogel could be formed at an acidic pH (∼6.5) *via* the peptide self-assembly.

### Oscillatory Rheology

ARES-RFS III rheometer (TA Instruments, United States) was employed to examine the oscillatory behavior of the peptide hydrogel. Prior to the measurement, the amphiphilic peptide solution (5 mg/mL) was prepared according to the method aforementioned and then transferred to the rheometer. After adjusting the solution pH to a value of ∼6.5, the storage modulus (G′) and loss modulus (G″) were recorded to understand the viscoelastic properties of the formed hydrogel.

### Transmission Electron Microscopy

The peptide hydrogel was diluted in DI water and the obtained diluted hydrogel solution was applied to a copper grid with Formvar film. After air-drying, the sample was visualized on a transmission electron microscope (TEM, Tecnai G^2^ Spirit BioTWIN).

### Circular Dichroism

JASCO J-815 spectropolarimeter was used to record the circular dichroism (CD) spectrum of the peptide hydrogel. Before the measurement, the peptide hydrogel was prepared in a 0.5 mm quartz cell. After placing the hydrogel in the spectropolarimeter, the conformation of the self-assembled peptide was analyzed with 4 s accumulations every 1 nm and averaged over three acquisitions.

### Fluorescence Spectroscopy

Fluorescence emission spectra were recorded on a Synergy HT multimode microplate reader with an excitation at 272 nm and emission data ranging between 280 and 600 nm.

### Synthesis of FITC-Labeled Polymyxin B

The FITC-labeled polymyxin B was synthesized based on the reaction between isothiocyanate group of FITC and the amino groups of polymyxin B. In brief, polymyxin B sulfate salt (20 mg, 0.014 mmol) was dissolved in the PBS solution (pH 7.4, 5 mL) and then FITC (8.6 mg, 0.022 mmol) dissolved in 0.5 mL of DMSO was added. After stirring in dark for 24 h, the mixture was transferred to a dialysis tube (MWCO 800) and dialyzed against DMSO for 24 h, followed by DI water for 48 h. The final dye-labeled polymyxin B (denoted FITC-polymyxin B) was collected after freeze-drying under vacuum.

### *In vitro* Drug Release

The *in vitro* release experiments were conducted at 37°C to evaluate the sustained release behavior of the peptide hydrogel. Prior to conducting the release experiment, the peptide hydrogel loading FITC-polymyxin B was prepared by dissolving 5 mg of amphiphilic peptide and 1 mg of FITC-polymyxin B in 1 mL of DI water at a pH of ∼10, followed by adjusting the solution pH to a neutral pH. The obtained hydrogel was placed in a cylindrical glass vial with only the top surface exposed for the drug release. Thereafter, 1 mL of PBS solution was added to the top of the peptide hydrogel. At a predetermined time interval, the total volume of PBS solution was removed and 1 mL fresh PBS solution was added after each sampling. The amount of FITC-polymyxin B released from the hydrogel was measured by examining the fluorescence intensity of FITC (*E*_*x*_ = 488 nm and *E*_*m*_ = 525 nm) using a microplate reader. The cumulative drug release was calculated as: Cumulative release (%) = (*M*_*t*_ *M*_∞_) × 100, where *M*_*t*_ is the amount of FITC-polymyxin B released from the peptide hydrogel at time *t* and *M*_∞_ is the amount of FITC-polymyxin B loaded in the peptide hydrogel.

### Cell and Bacteria Culture

NIH/3T3 fibroblasts were incubated in DMEM (pH 7.4) with 10% FBS at 37°C in a humidified atmosphere containing 5% CO_2_. Gram-negative bacteria MG1655 (*E. coli*) were incubated in LB medium at 37°C.

### *In vitro* Cytotoxicity

NIH/3T3 fibroblasts were used a model cell line to investigate the *in vitro* cytotoxicity of the amphiphilic peptides. The cells were seeded onto 96-well plates (5,000 cells per well) and then incubated in 100 μL of DMEM containing 10% FBS for 24 h. Thereafter, the medium was removed and the amphiphilic peptide dissolved in the medium was added. After 24 h incubation, the cells were washed with PBS solution and cell viability was measured using AlamarBlue assay according to the manufacturer’s protocol.

### Evaluation of Biocompatibility of Peptide Hydrogel

The amphiphilic peptide (5 mg) and polymyxin B (1 mg) were dissolved in 1 mL of DI water at a pH of 10. After filtration using a 200 μm membrane, the aqueous solution was transferred to a round disc and the hydrogel loading polymyxin B was prepared according to the method aforementioned. After gently washing the hydrogel using DMEM (3 × 2 mL) and then sterilization under UV irradiation for 30 min, NIH/3T3 fibroblasts (2 × 10^5^) suspended in 2 mL of DMEM with 10% FBS were seeded on the hydrogel surface. After incubation for 24 h, the medium was removed and PBS solution (2 mL, pH 7.4) containing 2 mM of calcein AM and 4 mM of EthD-1 was added for fluorescent live-dead staining assay. After incubation for 10 min, the stained cells were observed using a fluorescence microscope (Olympus). Viable cells were labeled by calcein AM with green fluorescence while apoptotic cells were stained by EthD-1 with red fluorescence. Viability was evaluated by measuring the percentage of viable cells of five randomly selected microscopic fields.

### Evaluation of Antibacterial Activity

The antibacterial activity was carried out according to previously reported methods (Zhao et al., 2017; Tian et al., 2020), in which the surface antibacterial activity of the hydrogel against *E. coli* was examined. In brief, the amphiphilic peptide (5 mg) and polymyxin B (1 mg) were dissolved in 1 mL of DI water at a pH of 10. After filtration using a 200 μm membrane, 0.5 mL of the peptide solution was added to a 24-well plate and the peptide hydrogel loading polymyxin B was prepared according to the protocol described above. After gently washing the hydrogel using LB medium (3 × 1 mL) and then sterilization under UV irradiation for 30 min, bacterial suspension (10 μL, 1 × 10^8^ CFU/mL) was added on the hydrogel surface. After incubation for 2 h, PBS solution (2 mL, pH 7.4) was used to sweep the bacteria out. Subsequently, the collected suspension of bacteria was diluted by 10^4^-fold and 50 μL of the resulting diluted suspension was transferred to an LB agar plate. After 12 h incubation, the colonies on the LB agar plate were counted. As a control, 10 μL of bacterial suspension (1 × 10^8^ CFU/mL) was added to a blank plate following the same steps described above. The average value of three independent experiments was collected.

## Results and Discussion

### Preparation and Characterizations of Peptide Hydrogels

**FIGURE 2:**
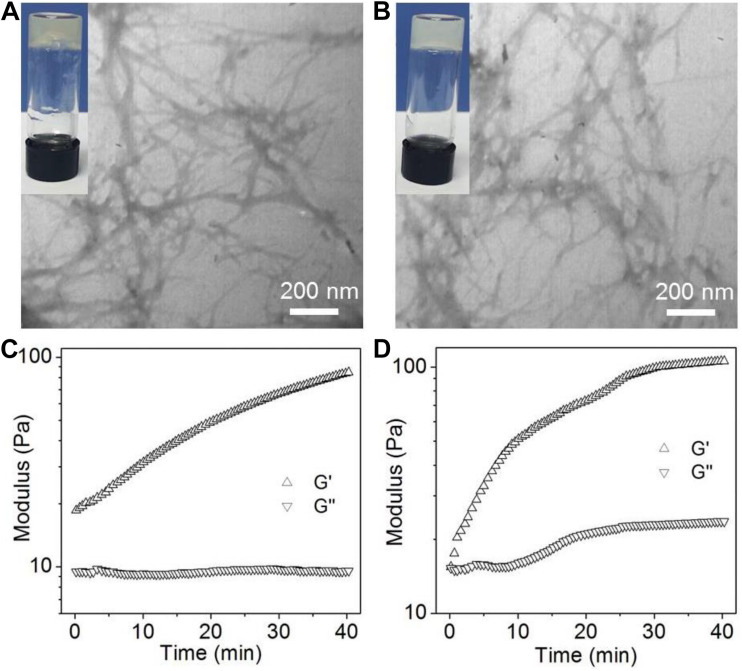
**(A,B)** Photo and TEM images of the supramolecular hydrogels self-assembled from peptide 1 **(A)** and 2 **(B)**. **(C,D)** Oscillatory rheology behaviors of the supramolecular hydrogels self-assembled from peptide 1 **(C)** and 2 **(D)**.

The structure of the amphiphilic peptide is shown in Figure 1. In this work, two amphiphilic peptides (denoted peptide 1 and 2) were designed to prepare supramolecular hydrogels. For these two peptides, they show a similar structure composed of a hydrophobic Nap tail and a hydrophilic peptide backbone. The hydrophobic Nap tail is expected to provide π-stacking interaction while the peptide backbone could afford hydrogen bonding interaction, which cooperate to drive the peptide self-assembly. In addition, considering that there are positively charged amino groups in the structure of polymyxin B (Figure 1), two carboxylic acid groups were incorporated into the structure of peptide 1, which is expected to form electrostatic attraction interaction to achieve sustained release of polymyxin B from the formed hydrogel. As shown in Figure 2, when dissolving these peptides into DI water at pH 10 followed by adding concentrated HCl solution, they could spontaneously self-assemble into stable supramolecular hydrogels (denoted Gel1 and Gel2) at an acidic pH (pH 6.5 for peptide 1 and pH 6.8 for peptide 2) within several minutes (Figures 2A,B). The viscoelastic properties (Figures 2C,D) show that the storage modulus (G′) is higher than the loss modulus (G″) of the formed hydrogels, which is a typical gel characteristic rheological behavior of solid-like materials (Xu et al., 2010, 2012). TEM measurement shows that the self-assembly of these two peptides induces the formation of uniform nanofibers (Figures 2A,B), which cross-link with each other to form the porous network of the formed hydrogel.

**FIGURE 3:**
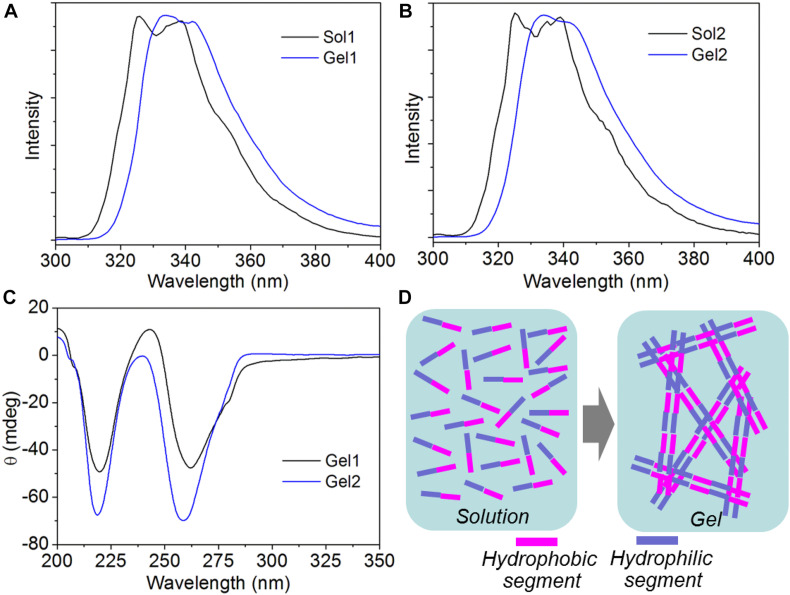
**(A,B)** Fluorescent emission spectra of the solution of peptide 1 [Sol1, **(A)**] and 2 [Sol2, **(B)**], and their self-assembled hydrogels (Gel1 and Gel2). **(C)** CD spectrum of the supramolecular hydrogels self-assembled from peptide 1 and 2. **(D)** Schematic illustration of the self-assembly of peptide 1 and 2 into supramolecular hydrogels.

Having validated the ability of the amphiphilic peptides to form stable hydrogels, we next investigated their self-assembly mechanism using CD and fluorescence spectroscopy. The fluorescent emission spectra of the amphiphilic peptides and their self-assembled hydrogels are shown Figures 3A,B. It can be found that both peptide 1 and 2 show two typical asymmetric emission peaks of Nap groups at ∼325 and 338 nm. After self-assembly into supramolecular hydrogels, the above two emission peaks shift to ∼331 and 344 nm, respectively. This slight red shift indicates the formation of π-stacking interaction among the hydrophobic Nap tails in the nanofibers (Yang et al., 2006; Xu et al., 2012). This π-stacking interaction is further confirmed by CD analysis. As displayed in Figure 3C, t, a negative broad bands from 240 to 300 nm corresponding to π-π^∗^ transition of the hydrophobic Nap tails (Yang et al., 2007; Xu et al., 2012) is observable in the CD spectra of the supramolecular hydrogels. Additionally, the negative band centered at ∼219 nm is a typical CD signal of polypeptide with a β-sheet conformation, suggesting the formation of a β-sheet-like superstructure among the peptide backbones of the amphiphilic peptides (Behanna et al., 2005; Yang et al., 2006; Xu et al., 2010). Combining the results of emission spectra and CD analysis, the self-assembly of the amphiphilic peptides designed in this work is mainly driven by π-stacking of the hydrophobic Nap tails and β-sheet-like arrangement of the peptide backbones *via* intermolecular hydrogen bonding interaction (Figure 3D).

### Evaluation of Drug Release Behavior

**FIGURE 4:**
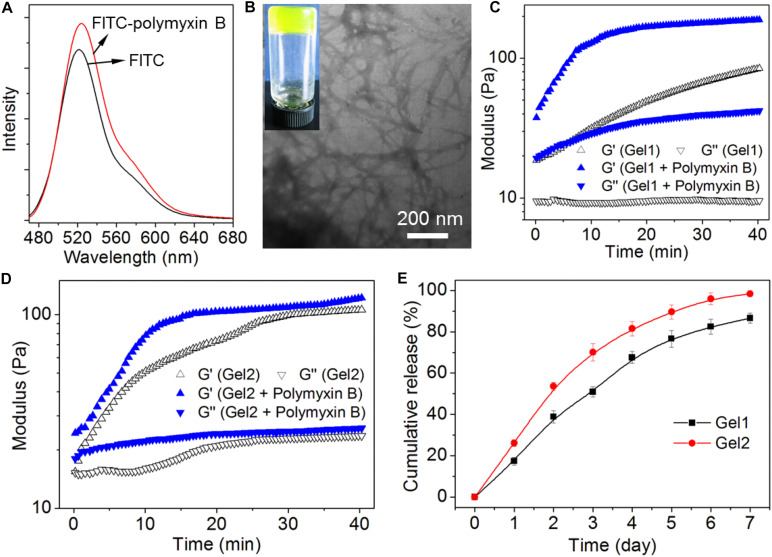
**(A)** Fluorescent emission spectrum of FITC-polymyxin B in aqueous solution. **(B)** Photo and TEM images of the hydrogel of peptide 1 (Gel1) loading FITC-polymyxin B. **(C,D)** Oscillatory rheology behaviors of Gel1 **(C)** and Gel2 **(D)** loading FITC-polymyxin B. **(E)** Cumulative release profile of polymyxin B encapsulated into Gel1 and Gel2 in PBS solution.

After investigating the peptide self-assembly behavior, we next evaluated the ability of the hydrogels of the amphiphilic peptides to encapsulate and release the antibiotic polymyxin B. Before the encapsulation, fluorescent dye FITC was used to label polymyxin B, which could facilitate the real-time tracking of polymyxin B release from the peptide hydrogels. Herein, fluorescent dye FITC was conjugated to polymyxin B *via* the reaction between the isothiocyanate group of FITC and the amino groups of polymyxin B. As shown in Figure 4A, the FITC-polymyxin B shows a typical signal at ∼525 nm, suggesting the successful conjugation of FITC to polymyxin B. After obtaining the dye labeled polymyxin B, we next encapsulated the FITC-polymyxin B into the hydrogel *via* mixing polymyxin B with the amphiphilic peptide in DI water followed by adding concentrated HCl aqueous solution. As shown in Figure 4B, the encapsulation of polymyxin B does not influence the self-assembly of the amphiphilic peptide and stable hydrogel with fibrous network could be obtained at a neutral pH, which is different from the acidic environment (pH ∼6.5) for the self-assembly of peptide 1, implying the formation of electrostatic attraction interaction between polymyxin B and peptide 1 (Behanna et al., 2005). As shown in Figure 1, because of the incorporation of two carboxylic acid groups in the structure of peptide 1, electrostatic repulsion interaction among the ionized peptide 1 molecules could be formed at a neural or basic environment and thus supramolecular self-assembly can be only achieved at an acidic pH (∼6.5). However, with the incorporation of polymyxin B into the self-assembly system, the positively charged polymyxin B could form electrostatic attraction interaction with the negatively charged peptide 1 at a neutral pH, which could thus drive the ionized peptide 1 molecules to get close to each other and subsequently drive the self-assembly of peptide 1 into supramolecular hydrogel *via* the π-stacking of the hydrophobic Nap tails and intermolecular hydrogen bonding interaction among the peptide backbones (Behanna et al., 2005; Xu et al., 2010). The electrostatic attraction interaction between polymyxin B and peptide 1 at a neutral pH was further confirmed by rheological behavior analysis (Figure 4C). Besides the intermolecular hydrogen bonding and π-stacking interactions (Figures 2A,C), the incorporation of polymyxin B could provide additional driving force for the self-assembly of peptide 1, thereby leading to significant increase in the mechanical strength of the resulting hydrogel (Xu et al., 2010). In contrast, due to the absence of electrostatic attraction, the incorporation of polymyxin B shows no obvious influence on the mechanical strength of the self-assembled hydrogel of peptide 2 (Figure 4D). With the electrostatic attraction interaction between polymyxin B and peptide 1, the resulting hydrogel shows an expected sustained release of the loaded antibiotic. Around 50% or 80% of loaded polymyxin B is released within 3 or 7 days (Figure 4E). In comparison, the release of polymyxin B from the hydrogel of peptide 2 is much faster and around 70 or 100% of the loaded polymyxin B has been released within 3 or 7 days.

### Evaluation of Biocompatibility and Antibacterial Activity

**FIGURE 5:**
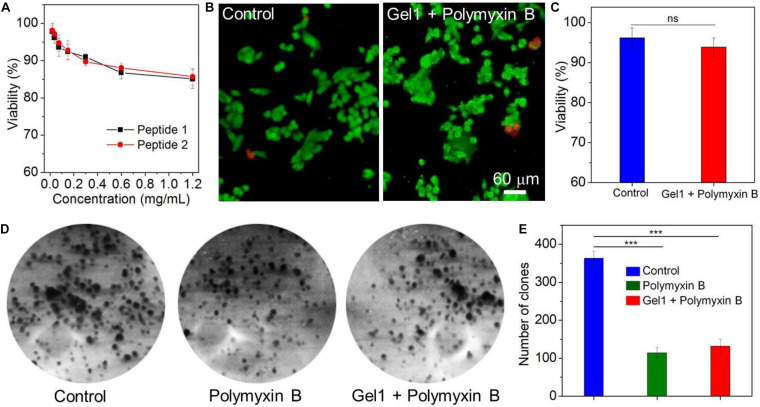
**(A)** Viability of NIH/3T3 fibroblasts incubated with the amphiphilic peptides at different concentrations for 24 h. **(B)** Live-dead assay of NIH/3T3 fibroblasts seeded in blank cell culture plate (Control) or on the surface of Gel1 loading polymyxin B followed by cultured with DMEM containing 10% FBS for 24 h. The live cells were stained with calcein AM as green fluorescence and dead cells were stained with EthD-1 as red fluorescence. **(C)** Viability of NIH/3T3 fibroblasts quantified from panel **(B)**. **(D,E)** Clone formation **(D)** and number of clones **(E)** of *E. coli* treated with free polymyxin B or Gel1 loading polymyxin B. *E. coli* incubated in blank culture plate was used control. **P < 0.01, ***P < 0.001.

Having validated the sustained release behavior of the peptide hydrogels, we finally chose Gel1 to evaluate its biocompatibility and antibacterial activity since this peptide hydrogel shows a stronger ability to control the release of polymyxin B compared to Gel2. The biocompatibility was assessed by examining cytotoxicity of the amphiphilic peptide against model cell line (NIH/3T3 fibroblasts) and proliferation of this cell line on the surface of Gel1 loading polymyxin B. As displayed in Figure 5A, the amphiphilic peptides designed in this work do not show apparent toxicity against NIH/3T3 fibroblasts. More than 85% of the cells are still alive after 24 h incubation with the peptide at a concentration of 1.2 mg/mL. Figure 5B shows the proliferation of NIH/3T3 fibroblasts on the surface of Gel1 loading polymyxin B. It can be seen that the cells could adhere and proliferate on the hydrogel surface. In comparison with the cells seeded in the cell culture plate, there is no obvious difference in the cell morphology and cell viability (Figure 5C), demonstrating the good biocompatibility of Gel1 loading polymyxin B. The antibacterial activity was evaluated by seeding the Gram-negative bacteria *E. coli* on the hydrogel surface followed by cultured an LB agar plate. As shown in Figures 5D,E, due to the release of polymyxin B from the peptide hydrogel to destroy *E. coli* membrane (Tsubery et al., 2000; Bhor et al., 2005), the formed clone number of *E. coli* is much less than the bacteria cultured in the blank plate, strongly demonstrating the good antibacterial activity of the peptide hydrogel loading polymyxin B. Notably, compared to the direct use of polymyxin B (∼30 μg/mL equivalent to the release of polymyxin B released from the hydrogel), although peptide hydrogel loading polymyxin B shows slightly weak ability to inhibit bacteria growth, the sustained polymyxin B release from the peptide hydrogel demonstrated in Figure 4E could facilitate to achieve long-term antibacterial activity.

## Conclusion

In summary, we herein designed a new amphiphilic peptide that could co-assemble with polymyxin B to form supramolecular hydrogel with antibacterial activity. In aqueous solution at neutral pH, electrostatic attraction interaction could be formed between the negatively charged amphiphilic peptide and positively charged polymyxin B, which could thus drive the ionized peptide molecules to get close to each other and subsequently trigger the self-assembly of the amphiphilic peptide into supramolecular hydrogel. More importantly, with the characteristic of sustained polymyxin B release, this supramolecular hydrogel shows an effective inhibition of bacteria growth. Combining its good biocompatibility, the supramolecular hydrogel developed herein could be used a new antibacterial candidate for the surgical site infection application.

## Data Availability Statement

The raw data supporting the conclusions of this article will be made available by the authors, without undue reservation.

## Author Contributions

HH and XX conceived and designed the experiments. LX, QS, and LH performed the experiments. LX, QS, LH, XX, and HH analyzed the data and co-wrote the manuscript. All authors contributed to the article and approved the submitted version.

## Conflict of Interest

The authors declare that the research was conducted in the absence of any commercial or financial relationships that could be construed as a potential conflict of interest.
